# Non-Enzymatic Glucose Sensor Composed of Carbon-Coated Nano-Zinc Oxide

**DOI:** 10.3390/nano7020036

**Published:** 2017-02-10

**Authors:** Ren-Jei Chung, An-Ni Wang, Qing-Liang Liao, Kai-Yu Chuang

**Affiliations:** 1 Department of Chemical Engineering and Biotechnology, National Taipei University of Technology (Taipei Tech), Taipei 10608, Taiwan; dai20020223@gmail.com (A.-N.W.); spa11012@hotmail.com (K.-Y.C.); 2 Department of Materials Physics, State Key Laboratory for Advanced Metals and Materials, University of Science and Technology Beijing, Beijing 100083, China; liao@ustb.edu.cn

**Keywords:** zinc oxide nanorod, carbon material, glucose, non-enzymatic electrochemical biosensor

## Abstract

Nowadays glucose detection is of great importance in the fields of biological, environmental, and clinical analyzes. In this research, we report a zinc oxide (ZnO) nanorod powder surface-coated with carbon material for non-enzymatic glucose sensor applications through a hydrothermal process and chemical vapor deposition method. A series of tests, including crystallinity analysis, microstructure observation, and electrochemical property investigations were carried out. For the cyclic voltammetric (CV) glucose detection, the low detection limit of 1 mM with a linear range from 0.1 mM to 10 mM was attained. The sensitivity was 2.97 μA/cm^2^mM, which is the most optimized ever reported. With such good analytical performance from a simple process, it is believed that the nanocomposites composed of ZnO nanorod powder surface-coated with carbon material are promising for the development of cost-effective non-enzymatic electrochemical glucose biosensors with high sensitivity.

## 1. Introduction

In recent years, biosensors have been investigated and applied in new drug discovery, clinical diagnosis, and forensic science. The goal is to minimize the size of detection instrument, detection sample, and detection period. The development of biosensors has become prevailingly studied topic in the biomedical field. In order to achieve specific detection, hybrid biosensors surface-modified with enzymes have been developed, called enzymatic sensors [1]. However, enzymatic sensors have drawbacks, including high cost, complicated production procedures, and short shelf lives, facing challenges in market. Additionally, an enzymatic sensor is impossible to implant into the human body for the long term and in situ monitoring due to the immobilized enzyme would degrade quickly [2]. In order to overcome this limitation, researchers place high expectations on non-enzymatic sensors; nevertheless, there are several critical issues, such as stability, selectivity, sensitivity, and detection limit [3,4,5].

Zinc oxide (ZnO) is a II–VI semiconductor with a wide direct band gap (3.37 eV) and electron binding energy (60 meV). Different nano-structures of ZnO can be controlled through various preparation processes to acquire the desired physical properties, such as vapor–liquid–solid (VLS), chemical vapor deposition (CVD), hydrothermal processes, solution–liquid–solid (SLS), and capping agents/surfactant-assisted synthesis [6,7,8,9]. In the past 20 years, the ZnO nanostructure-based sensors have seen growing importance placed on research of various sensor techniques from chemical and biosensors, to UV and pH sensors [10,11,12,13,14,15]. In this study, we focused on glucose biosensors because of the upstream health issue on diabetes [16,17,18]. For glucose sensing, enzymatic and non-enzymatic ZnO glucose sensors are commonly reported [19,20,21,22,23,24,25,26,27,28]. Here, we have summarized some of the latest and important studies in Table 1 and the sensing performances are listed. Due to the pH-sensitive glucose oxidase, most of the studies were performed in a neutral buffer solution (pH 6.5–7.5) and cannot be used in severe pH conditions. The enzymatic sensors usually possess low detection limits and excellent sensitivity; however, the linear detection range is below human blood glucose concentration, ranging from 4 to 6 mM. Nevertheless, the linear range can be extended by decorating ZnO with carbon material to enhance the electron conductivity, surface area, and biocompatibility [20,22]. High conductivity could play an important role in the direct electrochemistry. Generally, the non-enzymatic sensors show higher detection limits than the enzymatic ones. A large linear range and an excellent sensitivity are two great benefits for rapid and direct detection in blood samples. In this study, we propose carbon-coated ZnO to enhance the performance for direct glucose sensing. We prepared zinc oxide (ZnO) nanorod powder through a hydrothermal process, and then used a chemical vapor deposition method to surface-coat with carbon [29,30]. The ZnO and carbon-coated ZnO were immobilized onto a glassy carbon electrode to form non-enzymatic glucose sensors for comparison. Detailed investigations were then carried out.

## 2. Materials and Methods

**Figure 1:**
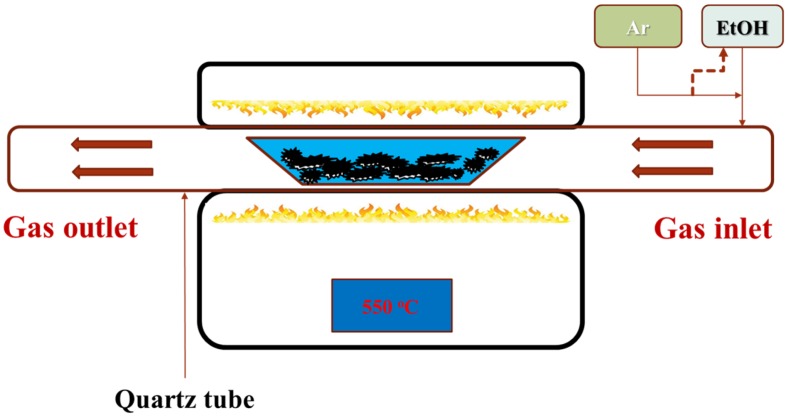
Scheme of the chemical vapor deposition (CVD) process.

In order to prepare the ZnO nanorod powder, 50 mL of 0.01 M Zn(NO_3_)_2_ was added dropwise into 50 mL 1 M NaOH for 15 min under stirring, and then the solution was heated to 95 °C and maintained for two hours. The precipitated ZnO nanorod powder was then centrifuged, thoroughly washed, and dried. The ZnO nanorod powder was then prepared for carbon coating. Carbon coating was performed using chemical vapor deposition (Figure 1). The source of carbon was ethanol carried by argon with a 50 sccm flow rate. The reaction was carried out at 550 °C for 1.5 h. Prior to the cyclic voltammetric (CV) analysis, the structure of the as-synthesized ZnO powder was characterized by X-ray diffraction and θ/2θ scan ranged from 20° to 70°, where the ZnO was identified according to joint committee on powder diffraction standards (JCPDS) card No. 36-1451. The structure of the carbon-coated (ZnO@C) and uncoated ZnO powders was observed from the scanning electron microscope (SEM) topographies, high-resolution transmission electron microscopy (HRTEM) images, and selected area electron diffraction (SAED) pattern. Raman spectroscopy was used to identify the carbon coating. 

The materials for sensor electrode were assembled on a 3 mm diameter glassy carbon working electrode (GC) (CHI104, CH Instrument, Inc., Austin, TX, USA). One milligram of carbon-coated ZnO (ZnO@C) nanorod powder was well mixed with 1 mL 5% Nafion (Sigma-Aldrich, Shanghai, China) solution. Afterward, 1 mL of the mixed solution was transferred onto the glassy carbon (GC) electrode and dried at room temperature for 10 min to serve as a working electrode for CV glucose detection. In comparison with the ZnO@C/GC electrode, ZnO on a glassy carbon electrode (ZnO/GC) was carried out using the abovementioned method. Hence, three different working electrodes, including ZnO@C/GC, ZnO/GC and bare glassy carbon (GC), were used. As for the reference and the auxiliary electrodes, the commercial Ag/AgCl reference electrode (RE-1B) and the platinum electrode were utilized, respectively. The CV scan was measured by utilizing a potentiostat CHI611E with a scanning range from +1 V to −1 V. First, the CV profiles of the three different working electrodes were assessed in 1 M NaOH_(aq)_ solution (pH = 12.5) and the similar solution with 1 mM glucose to demonstrate the analytical performance of ZnO and ZnO@C. To further investigate the ZnO@C/GC detector sensitivity and its limit, different scanning speeds from 10 mV/s to 100 mV/s, pH values from 4 to 12.5, and glucose concentrations from 0 to 11 mM were used. Three standard buffer solutions were facilitated for pH = 4, 7, and 10, and 1 M NaOH_(aq)_ was utilized for pH = 12.5. Furthermore, the current vs. time curve of the ZnO@C/GC electrode was measured at 0.38 V in 1 M NaOH_(aq)_ with respect to the glucose adding under a constant rate of 1 mM/sec, ranging from 1 to 14 mM, from which the corresponding current vs. glucose concentration was plotted and calibrated. Last, the amperometric response of ZnO@C/GC electrode to 1.0 mM glucose as well as 1 mM interferents of citric acid (CA), uric acid (UA), and dopamine (DA) was measured to investigate the specificity in glucose censing.

## 3. Results and Discussion

**Figure 2:**
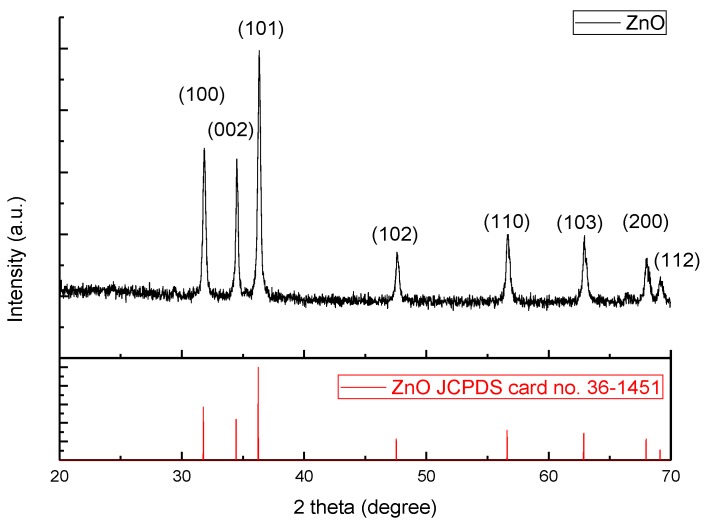
X-ray diffraction (XRD) pattern of the as-synthesized zinc oxide (ZnO) nanorod powder.

**Figure 3:**
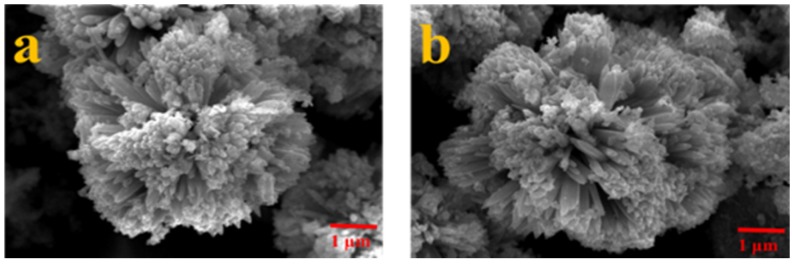
Micrographs of (**a**) ZnO and (**b**) ZnO (ZnO@C) nanorod powder.

The X-ray diffraction (XRD) pattern of the as-synthesized ZnO powder is in Figure 2 and it is identical to the JCPDS card as shown in the bottom of the figure, indicating a hexagonal close packing (HCP) ZnO structure. From the interplanar spacing of all (hkl) directions, the lattice parameter of the *a*-axis and *c*-axis can be estimated by combining Bragg’s law and the plane-spacing equation, and the resultant lattice parameters of *a*- and *c*-axes are 3.22 ± 0.02 Å and 5.21 ± 0.01 Å, respectively. Hence, the *c*/*a* ratio is 1.62, which is close to the ideal ZnO wurtzite structure (*c*/*a* = 1.633) [12]. The microstructures of the carbon-coated (ZnO@C) and uncoated ZnO nanoparticles were observed from the SEM image, as shown correspondingly in Figure 3a,b, in both of which ZnO nanorod structures can be found. Clearly, the carbon coating does not change the microstructure and only results in slightly larger ZnO particle size. 

**Figure 4:**
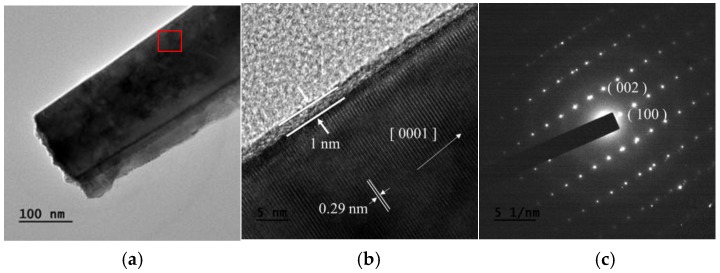
(**a**) Transmission electron microscopy (TEM) image; (**b**) high-resolution transmission electron microscopy (HRTEM) image; and (**c**) selected area electron diffraction (SAED) pattern of ZnO (ZnO@C) nanorod powder.

**Figure 5:**
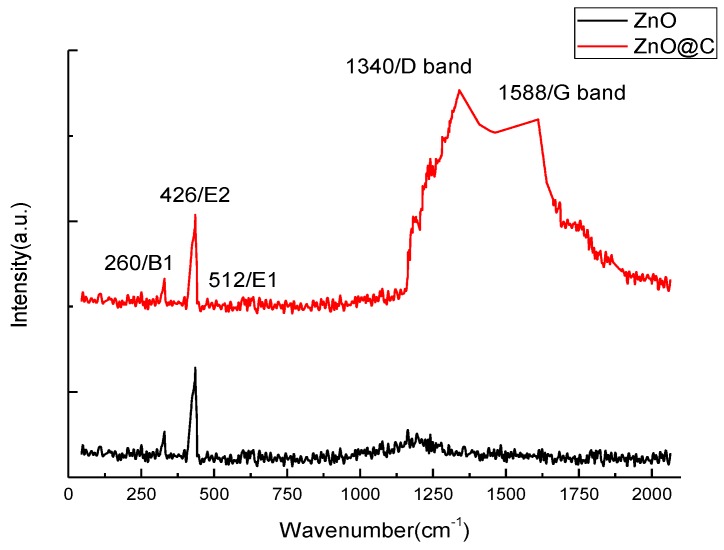
The Raman spectra of ZnO and carbon-coated ZnO nanorod powder. The Raman spectrum of ZnO was labeled according to the B1, E2, and E1 vibration modes, and those of carbon are D and G.

A more detailed structural observation of the carbon coating was made by the TEM and HRTEM images, corresponding to Figure 4a,b. A thin and uniform carbon layer of 1 nm was observed on the ZnO nanorods; and a preferential growth of basal plane (0001) [0001] was found. In addition, the SAED pattern (Figure 4c) shows that the ZnO nanorods are close to a single crystal. To analyze the thin carbon layer on ZnO, Raman spectroscopy was utilized and the comparison with uncoated ZnO is illustrated in Figure 5. The Raman spectrum of ZnO is labeled according to the B1, E2, and E1 vibration modes, where the E2 mode processing of 426 cm^−1^ was the most significant peak in both samples. A group of overlapping peaks corresponding to carbon D and G bands at 1340 cm^−1^ and 1588 cm^−1^ was observed. The intensity ratio (*I*_D_/*I*_G_) was 0.98, and this ratio corresponds to a tetrahedral amorphous carbon (ta-C) structure [31,32]. 

**Figure 6:**
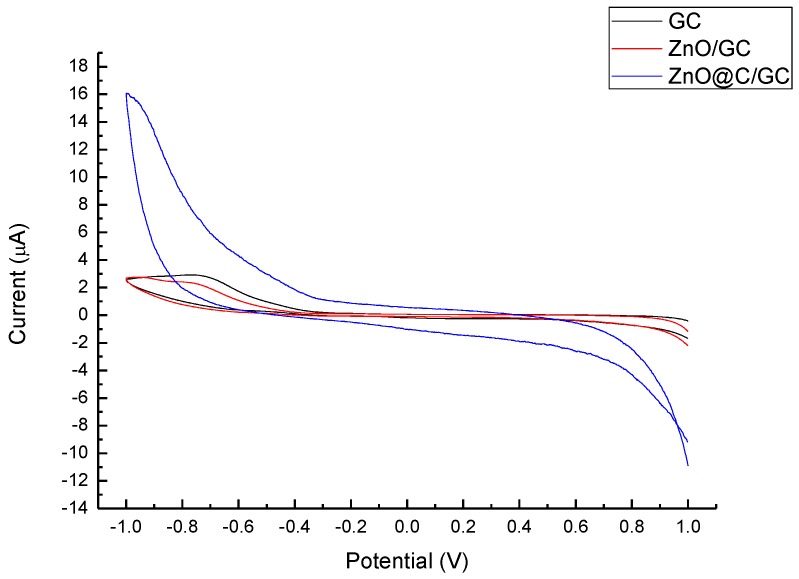
Cyclic voltammetric (CV) profiles of GC, ZnO/GC, and ZnO@C/GC electrodes in 1 M NaOH_(aq)_ solution.

**Figure 7:**
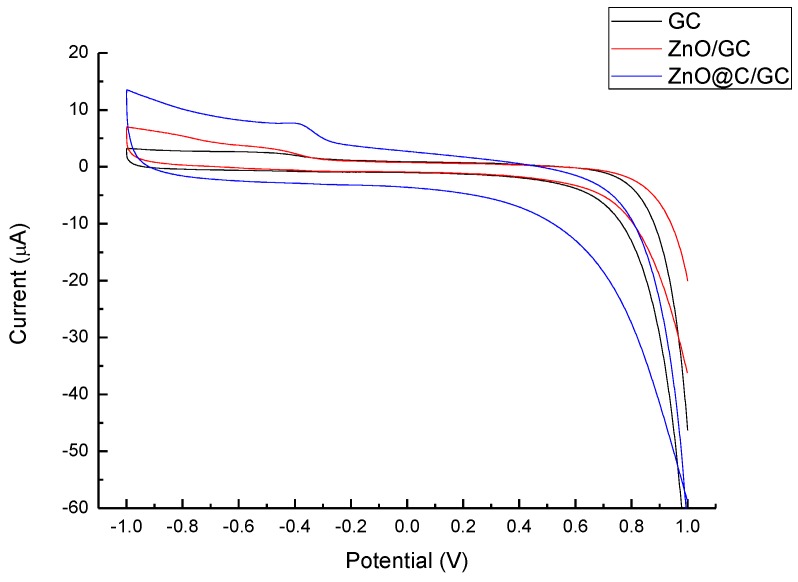
CV profiles of GC, ZnO/GC and ZnO@C/GC electrodes for 1 mM glucose sensing.

Figure 6 and Figure 7 depict the CV of the analytical performances of ZnO and ZnO@C in 1 M NaOH_(aq)_ and 1 mM glucose solutions, respectively. The ZnO@C electrode shows a great improvement of the sensitivity compared with the ZnO/GC and GC electrode in both solutions, while the ZnO/GC and GC electrode show similar CV curves in both situations. Evidently, the thin amorphous carbon layer (1 nm) is a key factor for the impressive improvement for sensing electrons and promoting the oxidation-redox reaction. Zhou et al. reported a similar ZnO nanowire or nanorod electrode for glucose sencing [28], for which the ZnO did not exhibit specific redox reaction with glucose. Without carbon decoration, ZnO does not react directly with glucose in the non-enzymatic detection. Hence, the role of ZnO in non-enzymatic sensors is inferred to accelerate the electron transfer. The carbon coating provides binding sites and electron transportation platforms for glucose.

**Figure 8:**
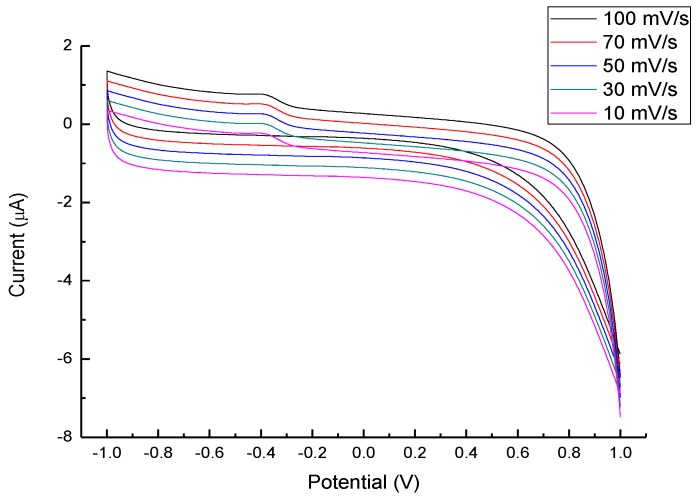
CV profiles under different scan rates in 1 M NaOH (aq) solution and 1 mM glucose.

**Figure 9:**
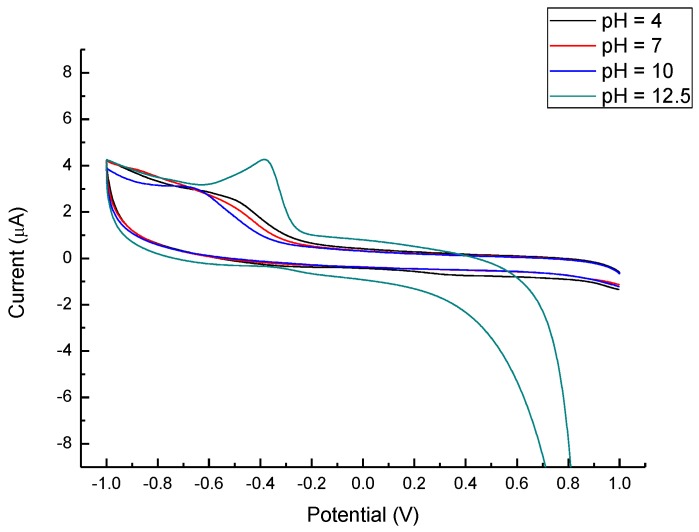
CV profile of ZnO@C/GC electrodes under different pH values.

In order to gain more understanding of the reaction and optimize the detection range, different scanning speeds (Figure 8) and pH values (Figure 9) were used on the CV scan of the ZnO@C/GC electrode. The symmetrical forward and backward current vs. the potential plots indicate that the redox reaction of glucose in 1 M NaOH_(aq)_ solution should be a reversible redox reaction. Moreover, the I_PC_ increased as the scanning rate increased from 10 mV/s to 100 mV/s, and the I_PC_ was proportional to the square root of the scan speed following the Randles–Sevcilk equation. By comparing with the current range, the pH = 12.5 in NaOH_(aq)_ is much more sensitive than rest of the pH values; still, more complete CV plots are needed and will be further studied in the future. At −0.4V, there was an additional peak from the enhanced redox reactions. The enhanced redox reaction was attributed to the great dependence of glucose oxidation on the influx of OH^−^ [25]. The weakly bound hydrogen atom at glucose is the first to be detached on electrodes at low potentials. Hence, the influx of OH^−^ might enhance glucose oxidation by neutralizing protons generated during the dehydrogenation step. At pH 12.5, the current of redox reactions became obvious, and the condition of −0.4 V, pH 12.5, and a 100 mV/s scan rate was selected as the optimized detection situation. In brief, the optimized glucose detection using the ZnO@C/GC electrode in this study is pH = 12.5 and a speed of 100 mV/s. The asymmetry in the CV curves, attributed to three major mechanisms, including electron transportation between glucose and electrode, complicated kinetic control and diffusion control. Similar results were also reported in [24,25,26,28,30]. 

**Figure 10:**
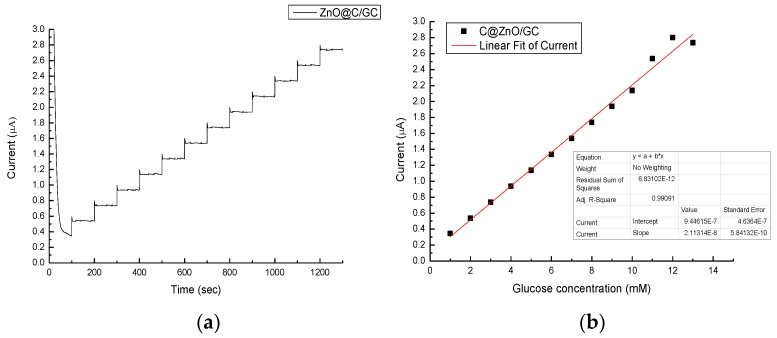
(**a**) The current vs. time curve of ZnO@C/GC electrode in 1 M NaOH_(aq)_ and glucose_(aq)_ solution, where the glucose_(aq)_ was added 1 mM per 100 s; (**b**) The corresponding linear fitting of the stabilized current and glucose concentration from (**a**).

**Figure 11:**
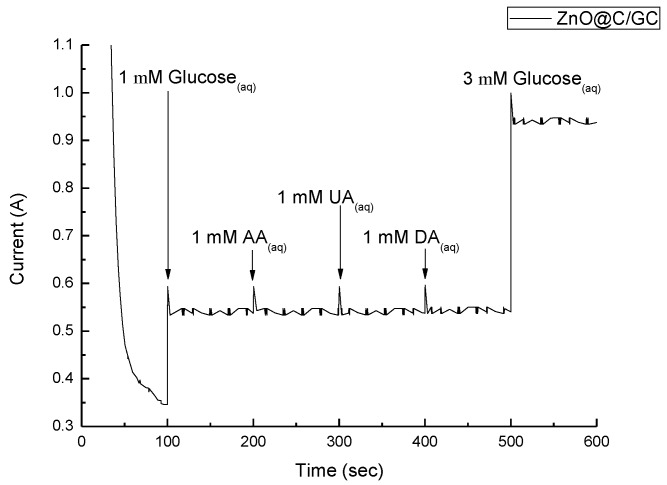
Amperometric response of ZnO@C/GC electrode to 1.0 µM glucose, as well as 1 mM interferents of citric acid (CA), uric acid (UA), and dopamine (DA).

With the optimized conditions for CV glucose detection, we performed the glucose sensitivity test using various concentrations, adding at a rate of 1 mM per 100 s (Figure 10). The current increased linearly with the increasing glucose concentration and the linear regression was calibrated in Figure 10b with the correlation factor of 0.99. A perfect linear region can be found up to 10 mM, which is right in the range of human blood (4–6 mM). Since blood contains of lot of different chemicals, the amperometric response of the ZnO@C/GC electrode was measured to investigate the specificity in glucose sensing (Figure 11). At 100 s 1 mM glucose was added and the current increased significantly, then stabilized until the next interferent was added. The other three interferents (CA, UA, and DA) were added at a time interval of 100 s, and the stabilized currents remained the same as the first glucose addition. In the last 100 s, 3 mM glucose was added. The current responded similarly to the first glucose addition, but with an increasingly stabilized current (about 1.7 times), which is slightly smaller than in Figure 10 (about 2.2 times). The three interferents might influence the current value, but do not affect the specificity of the glucose sensing. However, the pH value of human blood is normally around 7 and this means that the blood sample requires some pre-treatment to increase the pH value. Still, the glucose concentration using ZnO@C can detect, linearly, up to 10 mM, meaning no dilution of the sample is needed and, thus, the accuracy will not be influenced. Therefore, the ZnO@C holds great potential for non-enzymatic glucose sensor applications. The sensitivity was calculated to be 2.97 μA/cm^2^mM, as shown in Table 1, which is the most optimized ever reported. 

## 4. Conclusions 

In this study, we investigated a carbon-coated zinc oxide (ZnO@C) nanorod material for non-enzymatic glucose censing via a combination of hydrothermal synthesis and chemical vapor deposition. A series of tests, including crystallinity analysis, microstructure observation, and electrochemical property investigations, were carried out. The thin amorphous carbon layer (1 nm) is a key factor for the impressive improvement for sensing electrons and promoting the redox reaction. For cyclic voltammetric (CV) glucose detection, the optimized conditions for the proposed material are pH = 12.5 and a speed of 100 mV/s. Excellent analytical performance from the simple process include a low detection limit of 1 mM and a linear range from 1 mM to 13.8 mM. The ZnO nanorod powder surface-coated with carbon material is promising for the development of cost-effective non-enzymatic electrochemical glucose biosensors with high sensitivity. 

## Figures and Tables

**Table 1:** Important performance of enzymatic and non-enzymatic glucose sensors are summarized.

Sensor	Detect Limit (mM)	Linear Range (mM)	Sensitivity (μA/cm^2^mM)	Reference
**Enzymatic Glucose Sensor**				
GO*x*/ZnO/Au	10^−2^	0.01–3.45	23.1	Wei et al. 2006 [19]
GO*x*/C@ZnO nanowire/Ti	10^−3^	0.01–16	35.3	Liu et al. 2009 [20]
GO*x*/ZnO	5.6 × 10^−3^	Not mentioned	21	Ren et al. 2009 [21]
GR–CNT/ZnO–GO*x*	4.5 × 10^−3^	0.01–6.5	5.36	Hwa et al. 2014 [22]
GO*x*/BSA/Nafion/ZnO nanorod/GE	2.2 × 10^−4^	0.6–1.4	10.911	Marie et al. 2015 [23]
GO*x*/ZnO nanoparticle/IL/ESM	10^−10^	10^−9^–600	Not mentioned	Noor et al. 2015 [24]
GO*x*/Pt-Pb/CNTs	10^−3^	Up to 11	17.8	Cui et al. 2007 [25]
GO*x*/Cu/MWCNTs	2.1 × 10^−4^	0.7–3.5	251.4	Kang et al. 2007 [26]
**Non-enzymatic glucose sensor**				
ZnO nanoparticle	Not mentioned	1–10	38.133	Singh et al. 2012 [27]
ZnO–CuO	2.1 × 10^−4^	0.47 × 10^−^^3^–1.6	3066.4	Zhou et al. 2014 [28]
ZnO nanowire/EμPAD	5.95 × 10^−2^	0–15	8.24	Zhao et al. 2015 [29]
Ni/NiO-rGO-Nafion/SPE	1.8 × 10^−3^	0.03–6.44	1997	Zhang et al. 2016 [30]
C@ZnO/GC	1	1–13.8	2.97	This study
